# Clinical Antiviral Efficacy of Remdesivir in Coronavirus Disease 2019: An Open-Label, Randomized Controlled Adaptive Platform Trial (PLATCOV)

**DOI:** 10.1093/infdis/jiad275

**Published:** 2023-07-20

**Authors:** Podjanee Jittamala, William H K Schilling, James A Watson, Viravarn Luvira, Tanaya Siripoon, Thundon Ngamprasertchai, Pedro J Almeida, Maneerat Ekkapongpisit, Cintia Cruz, James J Callery, Simon Boyd, Orawan Anunsittichai, Maliwan Hongsuwan, Yutatirat Singhaboot, Watcharee Pagornrat, Runch Tuntipaiboontana, Varaporn Kruabkontho, Thatsanun Ngernseng, Jaruwan Tubprasert, Mohammad Yazid Abdad, Srisuda Keayarsa, Wanassanan Madmanee, Renato S Aguiar, Franciele M Santos, Elizabeth M Batty, Pongtorn Hanboonkunupakarn, Borimas Hanboonkunupakarn, Sakol Sookprome, Kittiyod Poovorawan, Mallika Imwong, Walter R J Taylor, Vasin Chotivanich, Chunlanee Sangketchon, Wiroj Ruksakul, Kesinee Chotivanich, Sasithon Pukrittayakamee, Arjen M Dondorp, Nicholas P J Day, Mauro M Teixeira, Watcharapong Piyaphanee, Weerapong Phumratanaprapin, Nicholas J White, for the PLATCOV Collaborative Group, Nicholas J White, William H K Schilling, Viravarn Luvira, James J Callery, Nicholas P J Day, Sasithon Pukrittayakamee, Simon Boyd, Cintia Cruz, Arjen M Dondorp, Walter R J Taylor, James A Watson, Watcharapong Piyaphanee, Kittiyod Poovorawan, Thundon Ngamprasertchai, Tanaya Siripoon, Borimas Hanboonkunupakarn, Kesinee Chotivanich, Podjanee Jittamala, Mallika Imwong, Maneerat Ekkapongpisit, Varaporn Kruabkontho, Thatsanun Ngernseng, Jaruwan Tubprasert, Mohammad Yazid Abdad, Srisuda Keayarsa, Orawan Anunsittichai, Maliwan Hongsuwan, Yutatirat Singhaboot, Wanassanan Madmanee, Elizabeth M Batty, Runch Tuntipaiboontana, Watcharee Pagornrat, Vasin Chotivanich, Wiroj Ruksakul, Chunlanee Sangketchon, Pongtorn Hanboonkunupakarn, Sakol Sookprome, Mauro M Teixeira, Pedro J Almeida, Renato S Aguiar, Franciele M Santos

**Affiliations:** Mahidol Oxford Tropical Medicine Research Unit, Bangkok, Thailand; Department of Tropical Hygiene, Faculty of Tropical Medicine, Mahidol University, Bangkok, Thailand; Mahidol Oxford Tropical Medicine Research Unit, Bangkok, Thailand; Centre for Tropical Medicine and Global Health, Nuffield Department of Medicine, University of Oxford, Oxford, United Kingdom; Mahidol Oxford Tropical Medicine Research Unit, Bangkok, Thailand; Centre for Tropical Medicine and Global Health, Nuffield Department of Medicine, University of Oxford, Oxford, United Kingdom; Department of Clinical Tropical Medicine, Faculty of Tropical Medicine, Mahidol University, Bangkok, Thailand; Department of Clinical Tropical Medicine, Faculty of Tropical Medicine, Mahidol University, Bangkok, Thailand; Department of Clinical Tropical Medicine, Faculty of Tropical Medicine, Mahidol University, Bangkok, Thailand; Clinical Research Unit, Centre for Advanced and Innovative Therapies, Belo Horizonte, Brazil; Mahidol Oxford Tropical Medicine Research Unit, Bangkok, Thailand; Mahidol Oxford Tropical Medicine Research Unit, Bangkok, Thailand; Centre for Tropical Medicine and Global Health, Nuffield Department of Medicine, University of Oxford, Oxford, United Kingdom; Mahidol Oxford Tropical Medicine Research Unit, Bangkok, Thailand; Centre for Tropical Medicine and Global Health, Nuffield Department of Medicine, University of Oxford, Oxford, United Kingdom; Mahidol Oxford Tropical Medicine Research Unit, Bangkok, Thailand; Centre for Tropical Medicine and Global Health, Nuffield Department of Medicine, University of Oxford, Oxford, United Kingdom; Mahidol Oxford Tropical Medicine Research Unit, Bangkok, Thailand; Mahidol Oxford Tropical Medicine Research Unit, Bangkok, Thailand; Department of Clinical Tropical Medicine, Faculty of Tropical Medicine, Mahidol University, Bangkok, Thailand; Mahidol Oxford Tropical Medicine Research Unit, Bangkok, Thailand; Mahidol Oxford Tropical Medicine Research Unit, Bangkok, Thailand; Mahidol Oxford Tropical Medicine Research Unit, Bangkok, Thailand; Mahidol Oxford Tropical Medicine Research Unit, Bangkok, Thailand; Mahidol Oxford Tropical Medicine Research Unit, Bangkok, Thailand; Mahidol Oxford Tropical Medicine Research Unit, Bangkok, Thailand; Centre for Tropical Medicine and Global Health, Nuffield Department of Medicine, University of Oxford, Oxford, United Kingdom; Department of Clinical Tropical Medicine, Faculty of Tropical Medicine, Mahidol University, Bangkok, Thailand; Mahidol Oxford Tropical Medicine Research Unit, Bangkok, Thailand; Department of Genetics, Ecology and Evolution, Institute of Biological Sciences, Universidade Federal de Minas Gerais, Belo Horizonte, Brazil; Department of Genetics, Ecology and Evolution, Institute of Biological Sciences, Universidade Federal de Minas Gerais, Belo Horizonte, Brazil; Mahidol Oxford Tropical Medicine Research Unit, Bangkok, Thailand; Centre for Tropical Medicine and Global Health, Nuffield Department of Medicine, University of Oxford, Oxford, United Kingdom; Bangplee Hospital, Ministry of Public Health, Samut Prakarn, Thailand; Mahidol Oxford Tropical Medicine Research Unit, Bangkok, Thailand; Department of Clinical Tropical Medicine, Faculty of Tropical Medicine, Mahidol University, Bangkok, Thailand; Bangplee Hospital, Ministry of Public Health, Samut Prakarn, Thailand; Mahidol Oxford Tropical Medicine Research Unit, Bangkok, Thailand; Department of Clinical Tropical Medicine, Faculty of Tropical Medicine, Mahidol University, Bangkok, Thailand; Mahidol Oxford Tropical Medicine Research Unit, Bangkok, Thailand; Department of Molecular Tropical Medicine and Genetics, Faculty of Tropical Medicine, Mahidol University, Bangkok, Thailand; Mahidol Oxford Tropical Medicine Research Unit, Bangkok, Thailand; Centre for Tropical Medicine and Global Health, Nuffield Department of Medicine, University of Oxford, Oxford, United Kingdom; Faculty of Medicine, Navamindradhiraj University, Bangkok, Thailand; Faculty of Science and Health Technology, Navamindradhiraj University, Bangkok, Thailand; Faculty of Medicine, Navamindradhiraj University, Bangkok, Thailand; Mahidol Oxford Tropical Medicine Research Unit, Bangkok, Thailand; Department of Clinical Tropical Medicine, Faculty of Tropical Medicine, Mahidol University, Bangkok, Thailand; Mahidol Oxford Tropical Medicine Research Unit, Bangkok, Thailand; Department of Clinical Tropical Medicine, Faculty of Tropical Medicine, Mahidol University, Bangkok, Thailand; Mahidol Oxford Tropical Medicine Research Unit, Bangkok, Thailand; Centre for Tropical Medicine and Global Health, Nuffield Department of Medicine, University of Oxford, Oxford, United Kingdom; Mahidol Oxford Tropical Medicine Research Unit, Bangkok, Thailand; Centre for Tropical Medicine and Global Health, Nuffield Department of Medicine, University of Oxford, Oxford, United Kingdom; Clinical Research Unit, Centre for Advanced and Innovative Therapies, Belo Horizonte, Brazil; Department of Clinical Tropical Medicine, Faculty of Tropical Medicine, Mahidol University, Bangkok, Thailand; Department of Clinical Tropical Medicine, Faculty of Tropical Medicine, Mahidol University, Bangkok, Thailand; Mahidol Oxford Tropical Medicine Research Unit, Bangkok, Thailand; Centre for Tropical Medicine and Global Health, Nuffield Department of Medicine, University of Oxford, Oxford, United Kingdom

**Keywords:** COVID-19, SARS-CoV-2, antiviral efficacy, pharmacometrics, remdesivir

## Abstract

**Background:**

Uncertainty over the therapeutic benefit of parenteral remdesivir in coronavirus disease 2019 (COVID-19) has resulted in varying treatment guidelines.

**Methods:**

In a multicenter open-label, controlled, adaptive, pharmacometric platform trial, low-risk adult patients with early symptomatic COVID-19 were randomized to 1 of 8 treatment arms including intravenous remdesivir (200 mg followed by 100 mg daily for 5 days) or no study drug. The primary outcome was the rate of severe acute respiratory syndrome coronavirus 2 (SARS-CoV-2) clearance (estimated under a linear model fit to the daily log_10_ viral densities, days 0–7) in standardized duplicate oropharyngeal swab eluates, in a modified intention-to-treat population. This ongoing adaptive trial is registered at ClinicalTrials.gov (NCT05041907).

**Results:**

The 2 study arms enrolled 131 patients (remdesivir n = 67, no study drug n = 64) and estimated viral clearance rates from a median of 18 swab samples per patient (a total of 2356 quantitative polymerase chain reactions). Under the linear model, compared with the contemporaneous control arm (no study drug), remdesivir accelerated mean estimated viral clearance by 42% (95% credible interval, 18%–73%).

**Conclusions:**

Parenteral remdesivir accelerates viral clearance in early symptomatic COVID-19. Pharmacometric assessment of therapeutics using the method described can determine in vivo clinical antiviral efficacy rapidly and efficiently.

## Teaser

In early symptomatic COVID-19, intravenous remdesivir accelerated viral clearance compared with no treatment.

Remdesivir has been used extensively as a parenteral treatment for coronavirus disease 2019 (COVID-19) in some regions, although therapeutic recommendations have varied widely [1–3]. The initial clinical trials of remdesivir suggested clinical benefit in reduced duration of hospitalization but did not demonstrate an in vivo antiviral effect. Until early 2022 the World Health Organization (WHO) recommended against use of remdesivir in treatment, largely based on lack of mortality reduction in the interim results from the WHO's multinational Solidarity trial [4, 5], and many countries did not license remdesivir for use. The WHO recommendation against the use of remdesivir has now been reversed, with updated results from the WHO's Solidarity trial indicating a small benefit [6, 7]. It is now appreciated that antiviral medications are more effective early in COVID-19 infections when viral burdens are highest, and they provide less benefit later in the course of illness in hospitalized patients where anti-inflammatory interventions show life-saving benefit [8, 9]. In the majority of cases, hospitalization occurs after approximately 1 week from symptom onset (this was the population studied in the Solidarity trial)—administration of remdesivir early in the course of infection has been shown to prevent progression to severe disease in high-risk outpatients [10, 11].

Large randomized controlled trials have since demonstrated the clinical efficacy of multiple small molecule drugs in the treatment of early COVID-19, notably molnupiravir and ritonavir-boosted nirmatrelvir [12–14]. These oral medications are simpler to administer compared with intravenous remdesivir. However, drug–drug interactions, other contraindications, and availability of these oral medications mean that remdesivir retains clinical utility in select populations [15]. Until now there has been no standardized method for assessing in vivo antiviral effects. Time to viral clearance is an insensitive measure that is highly dependent on baseline viral loads [16]. The PLATCOV study (Finding treatments for COVID-19: a phase 2 multi-centre adaptive platform trial to assess antiviral pharmacodynamics in early symptomatic COVID-19) is a platform randomized trial aiming to provide a standardized assessment in vivo of candidate antiviral drugs by measuring rates of viral clearance [17]. We report the antiviral activity of remdesivir in adults with early symptomatic COVID-19. The previously published clinical trials on remdesivir were conducted largely in unvaccinated patients infected with the early SARS-CoV-2 viral variants, which were more likely to result in hospitalization and severe outcomes than those prevalent today [1–3, 18–21]. This study was conducted in largely vaccinated individuals in a period that spanned the Delta and early Omicron variants.

## METHODS

PLATCOV is an ongoing phase 2 open-label, randomized, controlled, adaptive, pharmacometric platform trial (ClinicalTrials.gov identifier NCT05041907). It provides a standardized quantitative comparative method for in vivo assessment of potential antiviral treatments in low-risk adults with early symptomatic COVID-19. The primary outcome measure is the viral clearance rate derived from the slope of the log_10_ oropharyngeal viral clearance curve over the first 7 days following randomization, estimated under a linear model [16, 17]. The treatment effect is defined as the multiplicative change in viral clearance rate relative to the control arm (no study drug). The trial was conducted in the Faculty of Tropical Medicine, Mahidol University, Bangkok; Bangplee Hospital, Samut Prakarn; and Vajira Hospital, Navamindradhiraj University, Bangkok, all in Thailand, and in testing centers in Belo Horizonte, Minas Gerais, Brazil (see Supplementary Materials). All patients provided fully informed written consent. PLATCOV was coordinated and monitored by the Mahidol Oxford Tropical Medicine Research Unit (MORU) in Bangkok and was overseen by a trial steering committee, and its results were reviewed regularly by a data and safety monitoring board (DSMB).

### Randomization and Interventions

Randomization was performed via a centralized web application designed by MORU software engineers using RShiny, hosted on a MORU webserver. The control arm comprised a minimum proportion of 20% of patients with uniform randomization ratios applied across the treatment arms. All patients received standard symptomatic treatment. Remdesivir (Covifor: Hetero Drugs Ltd, Hyderabad, India [in Thailand, n = 58], and Veklury: Gilead Sciences, Foster City, California [in Brazil, n = 9]) was given by rate-controlled intravenous infusion over 60 minutes (reconstituted and added to 250 mL 0.9% saline) in an initial adult dose of 200 mg, followed by 100 mg once daily for 4 days to complete a 5-day course (Supplementary Materials). During this period, other patients were randomized to ivermectin (Thailand only, until 22 April 2022), casirivimab/imdevimab (Thailand only), favipiravir, nitazoxanide (Brazil only), fluoxetine (Thailand only from 1 April 2022), molnupiravir (Thailand only, from 6 June 2022), or nirmatrelvir/ritonavir (Thailand only, from 6 June 2022).

### Participants and Procedures

Previously healthy adults aged between 18 and 50 years were eligible for enrollment if they had early symptomatic COVID-19 (i.e., reported symptoms for ≤4 days), oxygen saturation ≥96%, were unimpeded in activities of daily living, and gave fully informed consent. SARS-CoV-2 positivity was defined either as a nasal lateral flow antigen test that became positive within 2 minutes (STANDARD Q COVID-19 Ag Test, SD Biosensor, Suwon-si, Korea) or a positive polymerase chain reaction (PCR) test within the previous 24 hours with a cycle threshold value <25 (all viral gene targets), both suggesting high viral loads. Exclusion criteria included taking any potential antivirals or preexisting concomitant medications, chronic illness or significant comorbidity, hematological or biochemical abnormalities, pregnancy (a urinary pregnancy test was performed in females), breastfeeding, or contraindication or known hypersensitivity to any of the study drugs.

Enrolled patients were either admitted to the study ward (in Thailand) or followed as outpatients at home (in Brazil). After randomization and baseline procedures (Supplementary Materials), oropharyngeal swabs (2 swabs from each tonsil) were taken as follows. A flocked swab (Thermo Fisher MicroTest and later COPAN FLOQSwabs) was rotated against the tonsil through 360° 4 times and placed in Thermo Fisher M4RT viral transport medium (3 mL). Swabs were transferred at 4°C–8°C, aliquoted, and then frozen at −80°C within 48 hours. Separate swabs from each tonsil were taken once daily from day 0 to day 7, and on day 14. Vital signs were recorded 3 times daily and symptoms and any adverse effects were recorded daily.

The TaqCheck SARS-CoV-2 Fast PCR Assay (Applied Biosystems, Thermo Fisher Scientific, Waltham, Massachusetts) quantitated viral loads (RNA copies/mL). This multiplexed real-time PCR method detects the SARS-CoV-2 N and S genes, and human RNase P in a single reaction. RNase P helped correct for variation in sample human cell content. Viral loads were quantified against ATCC heat-inactivated SARS-CoV-2 (VR-1986HK strain 2019-nCoV/USA-WA1/2020) standards. The lower limit of detection (LLOD) of the assay is approximately 50 copies/mL, and the lower limit of quantification (LLOQ) is approximately 200 copies/mL. Viral variants were identified using Whole Genome Sequencing (Supplementary Materials). Adverse events were graded according to the Common Terminology Criteria for Adverse Events version 5.0. Summaries were generated if the adverse event was grade 3 or higher and was new, or had increased in intensity. Serious adverse events were recorded separately and reported to the DSMB.

### Outcome Measures and Statistical Analysis

The primary outcome measure was the rate of viral clearance, expressed as a slope coefficient [16], and estimated under a Bayesian hierarchical linear model fitted to the daily log_10_ viral load measurements between days 0 and 7 (18 measurements per patient), using weakly informative priors and treating nondetectable viral loads (cycle threshold value ≥40) as left censored (see Supplementary Materials). The viral clearance rate (i.e., slope coefficient from the model fit) can be expressed as a clearance half-life (t_1/2_ = log_10_ 0.5/slope). The treatment effect was defined as the multiplicative change (%) in the viral clearance rate relative to the control arm (i.e., how much the test treatment accelerates viral clearance) [16]. A 50% increase in clearance rate thus equals a 33% reduction in clearance half-life. All cause hospitalization for clinical deterioration (until day 28) was a secondary endpoint. For each studied intervention, the sample size was adaptive based on prespecified futility and success stopping rules (details given in the Supplementary Materials).

All analyses were done in a modified intention-to-treat (mITT) population, comprising patients who had ≥3 days follow-up data. A sensitivity analysis showed excellent agreement between all the models (Supplementary Materials). Model fits were compared using approximate leave-one-out comparison as implemented in the package *loo*. All data analysis was done in R version 4.0.2. Model fitting was done in *stan* via the *rstan* interface. All code and data are openly accessible via GitHub: https://github.com/jwatowatson/PLATCOV-remdesivir.

## RESULTS

**Figure 1.**
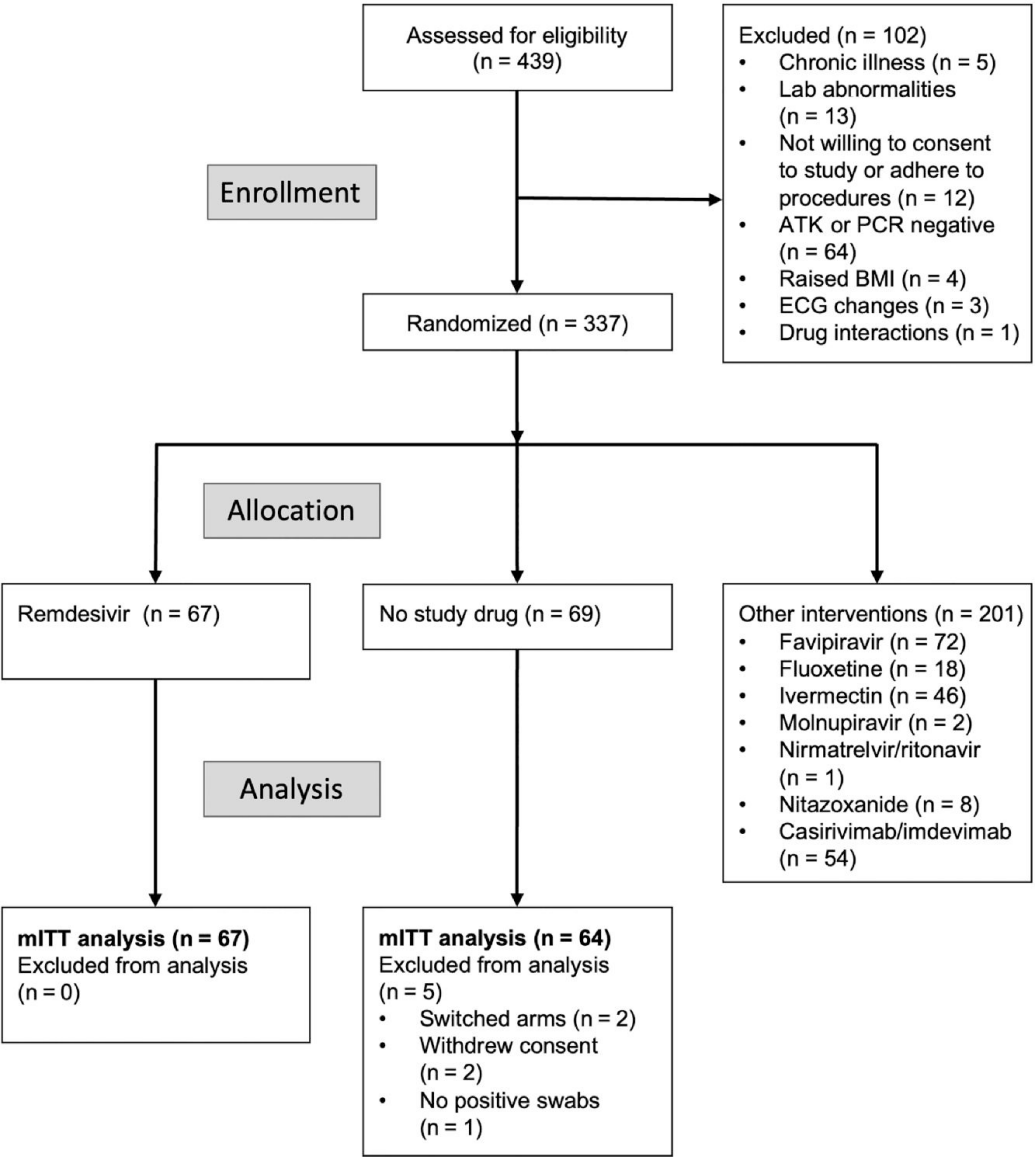
Consolidated Standards for Reporting Trials diagram of the PLATCOV phase 2 open-label, randomized, controlled adaptive platform trial for remdesivir in Thailand and Brazil. Enrollment ended 10 June 2022. Abbreviations: ATK, antigen test kit; BMI, body mass index; ECG, electrocardiogram; mITT, modified intention-to-treat; PCR, polymerase chain reaction.

The trial began recruitment on 30 September 2021. On 10 June 2022, remdesivir enrollment was stopped as the prespecified success margin had been reached. Of the 439 patients screened by that time, 337 were randomized to either remdesivir (67 patients), no study drug (69 patients), or to other interventions (201 patients) [17]. Five patients were excluded from the analyses (Figure 1), resulting in an mITT population of 131 patients (67 remdesivir and 64 no study drug) (Table 1). The total number of quantitative PCR (qPCR) viral density estimates in the analysis population from the first 8 days was 2356 (86% [2016/2356]) above the LLOD, with a median of 18 swabs taken per patient (range, 16–18). No patients developed severe COVID-19 but 1 patient in the control arm was hospitalized (see “Adverse Effects”).

### Virological Responses

**Figure 2.**
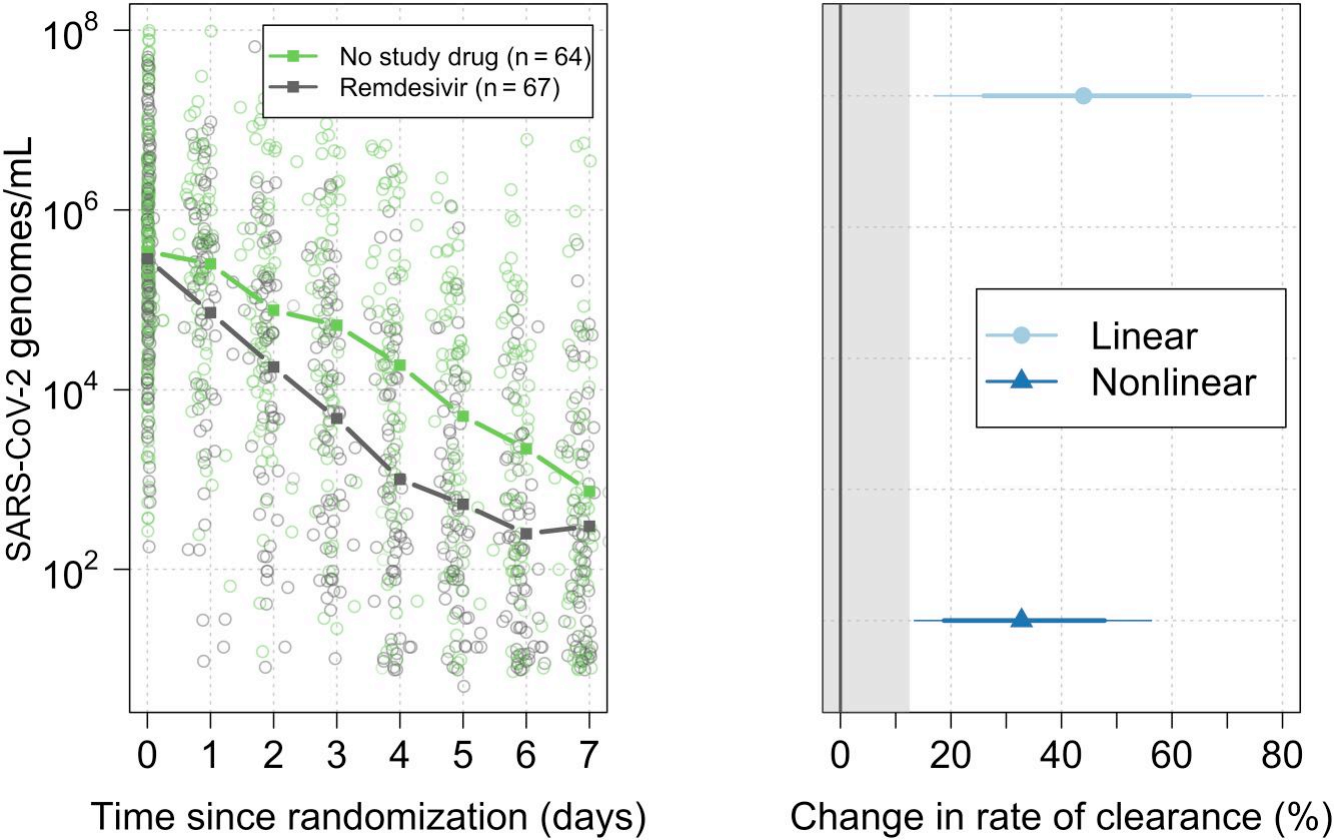
Effect of parenteral remdesivir on severe acute respiratory syndrome coronavirus 2 (SARS-CoV-2) viral clearance. Left: Median SARS-CoV-2 oropharyngeal virus clearance profiles following remdesivir (darker/grey) and no study drug (lighter/green). Right: estimated treatment effects under the linear model (light with circle) and nonlinear model (dark with triangle). The thick lines show the 80% credible interval (CrI); the thin lines show the 95% CrI.

**Figure 3.**
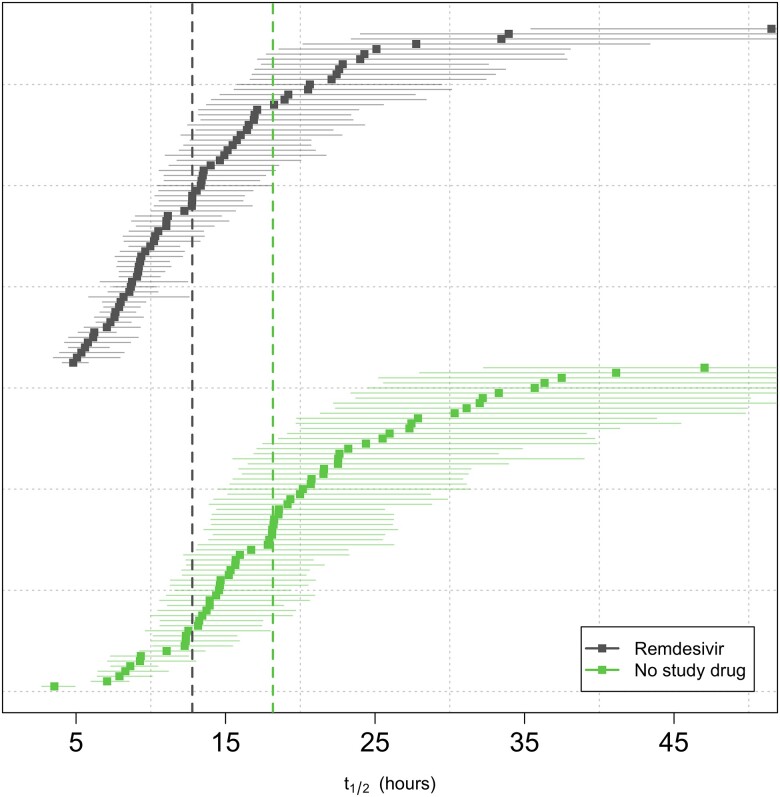
Individual estimate of the viral clearance half-lives with point estimates (squares) and 80% credible intervals (lines). The vertical dashed lines show the median population viral clearance half-lives by treatment arm (lower: no study drug; upper: remdesivir). The median estimated viral clearance half-lives under the linear model were 12.8 (range, 4.8–50.0) hours in the remdesivir arm and 18.0 (range, 3.6–46.7) hours in the contemporaneous control arm.

All analytical models of oropharyngeal virus clearance were in excellent agreement. Point estimates and credible intervals (CrIs) were similar, although the nonlinear model gave slightly smaller effect size estimates (Figure 2). In general, the nonlinear model (which allows some patients to have viral load increases after randomization) fitted the data better. All effect sizes reported in the main text are inferred under the prespecified main model: a linear model with intercept and slope adjusted for site and virus variant (this was also used in the interim analyses to make stopping decisions) (Supplementary Figure S2). The baseline geometric mean oropharyngeal viral load was 3.5 × 10^5^ RNA copies/mL (interquartile range, 5.9 × 10^4^ to 2.5 × 10^6^). Relative to the control arm, clearance of oropharyngeal virus in patients randomized to remdesivir was 42% faster (95% CrI, 18%–73%; probability of >12.5% acceleration: 0.99), Figure 2. The median estimated viral clearance half-lives under the linear model were 12.8 (range, 4.8–50.0) hours in the remdesivir arm and 18.0 (range, 3.6–46.7) hours in the contemporaneous control arm; that is, median virus clearance half-life was shortened by approximately one-third (Figure 3).

### Adverse Effects

The oropharyngeal swabbing and all treatments were well-tolerated. There were 3 serious adverse events (SAEs) in the control arm and 1 SAE in the remdesivir arm (Supplementary Tables S1 and S2). Two patients in the control arm and 1 in the remdesivir arm had asymptomatic raised creatinine phosphokinase levels (>10 times upper limit of normal) attributed to COVID-19–related skeletal muscle damage. This improved with fluids and supportive management and was considered unrelated to treatment. One patient (control arm) was readmitted 1 day after discharge because of chest pain and lethargy. All clinical and laboratory investigations were normal and the patient was discharged the following day. There were no treatment-related SAEs.

## DISCUSSION

This comparative in vivo pharmacodynamic assessment shows that remdesivir accelerates viral clearance in early COVID-19, as would be expected from an effective antiviral drug. Continued uncertainty over the efficacy and value of different COVID-19 treatments has resulted in substantial variation in therapeutic guidelines and clinical practice across the world. From 20 November 2020 until 22 April 2022, the 8 successive versions of the WHO's “Therapeutics and COVID-19: Living Guideline” gave a conditional recommendation *against* the use of remdesivir. Meanwhile, remdesivir continued to have Emergency Use Authorization status from the United States Food and Drug Administration and was used widely. It is estimated that 10 million people have been treated with remdesivir [22]. The first clinical trials, conducted in patients hospitalized with COVID-19 early in the pandemic, showed that administration of remdesivir was associated with shortening of the duration of hospitalization [1–3]. In 1 trial, the 5-day regimen demonstrated statistically significant benefit whereas the 10-day regimen did not [3], but in a separate trial there was no difference between the 5- and 10-day regimens [18]. The first large (N = 1062) double-blind, randomized, placebo-controlled trial of the 10-day regimen also suggested a reduction in mortality [2]. These trials were conducted early in the pandemic before general vaccine availability, at a time when hospitalization rates and mortality were high. Enthusiasm for remdesivir was then tempered in late 2020 by the negative preliminary results of the large, WHO-led, multicenter multinational open-label Solidarity trial in hospitalized patients [4]. Death occurred in 301 of 2743 patients receiving remdesivir and in 303 of 2708 receiving its control (rate ratio, 0.95 [95% confidence interval, .81–1.11]; *P* = .50). The open-label randomized platform trial DisCoVeRy conducted in hospitalized hypoxemic adult patients also failed to show benefits from remdesivir [19]. In contrast, the final results of Solidarity published in May 2022, which included a meta-analysis of all earlier trials, concluded that although remdesivir provided no benefit in ventilated patients, it did have a small benefit in protecting against death and progression to ventilation (or both) among the other hospitalized patients [7]. Other trials in hospitalized patients found modest effects from remdesivir in shortening hospital stay or avoidance of mechanical ventilation, but without reduction in mortality [20, 21].

It has become increasingly clear that antivirals are more effective in early COVID-19, when viral burdens are highest, than in late-stage disease (i.e., in hospitalized patients requiring oxygen or ventilation) where viral burdens have declined, inflammatory processes dominate, and anti-inflammatory interventions are most effective [8, 9]. This suggests that patients treated early in the course of their illness would derive greater proportional benefit from an antiviral medication. This is consistent with therapeutic results from other effective antiviral interventions [13, 14]. In the more recent randomized controlled trial of short-course remdesivir conducted in high-risk outpatients with early (i.e., <7 days illness) COVID-19 (PINETREE study), remdesivir significantly reduced the risk of disease progression [10]. In the 562 patients enrolled, hospitalization or death from any cause occurred in 2 patients (0.7%) in the remdesivir group and 15 (5.3%) in the placebo group (hazard ratio, 0.13 [95% confidence interval, .03–.59]).

In early COVID-19 illness viral load decay from the nasopharynx and oropharynx is approximately log-linear, although there is substantial interindividual variation in rates of clearance and baseline viral loads. The approximate 40% average acceleration in viral clearance rate observed with remdesivir in this study is similar in magnitude to that observed in trials with the oral drug molnupiravir, although the assessment methodologies were different [12, 13]. In the present study there was no evidence that antiviral efficacy differed between the viral variants. It also confirms antiviral efficacy in vaccinated populations. The considerable intraindividual variability in nasopharyngeal (or oropharyngeal) viral loads results in a low signal-to-noise ratio [16]. Frequent sampling is therefore required to characterize clearance rates. This may explain why studies that sampled infrequently, such as PINETREE [10], did not associate increased viral clearance with therapeutic efficacy. The method of calculation is also a factor. The PINETREE study estimated for each patient the difference between their baseline viral load (day 0) and the area under the viral time curve (AUC) based on 3 timepoints (days 2, 3, and 7). AUC was calculated using the trapezoid rule, and viral concentrations below the LLOQ were imputed as half the LLOQ. If viral clearance is truly rapid, this imputation approach biases toward a slower estimate (ie, closer to a no treatment response). Frequent viral load estimation allowed a different approach: fitting a hierarchical linear model to the serial viral load measurements and derivation of a rate constant (and thus elimination half-life). This method treated undetectable viral loads as left censored (ie, an unknown value below the limit of detection) and borrowed information across timepoints and patients. Another possible contributor to differences between this and earlier studies is the use of nasal, as opposed to oropharyngeal, swabbing. A study in rhesus macaques did not show high remdesivir concentrations in nasal epithelium, and there was prolonged viral shedding in these tissues [23].

This study has several limitations. The relationship between rate of viral clearance and therapeutic efficacy has not been well established, although across tested antiviral interventions there is a general direct relationship between acceleration of viral clearance and prevention of disease progression [12–14, 24, 25] (as there is for most acute infections). We intentionally evaluated the COVID-19 antiviral interventions in low-risk adults with high viral burdens in order to optimize the comparative assessment of the different drugs, and not in high-risk patients or the elderly, who are at greatest risk of disease progression. We assessed a 5-day course of remdesivir, although 3-day courses are also licensed in some countries. Another important limitation is that this is an open-label study, which may have led to more withdrawals in the control arm or bias in reporting adverse events or symptoms, although within this small study there were no safety concerns and the remdesivir was well-tolerated. Time to symptom resolution and time to fever clearance are shown in the Supplementary Figures S4 and S5. The differences do not reach statistical significance.

Remdesivir continues to have a role in the treatment of COVID-19 for certain populations [15]. The simple pharmacometric methodology presented demonstrates the in vivo antiviral efficacy of remdesivir and is readily performed anywhere where accurate qPCR viral quantitation can be performed. Duplicate daily oropharyngeal swabs are well-tolerated over 1 week (whereas daily nasopharyngeal swabbing is not) and they provide viral load measurements from which robust estimates of viral clearance can be obtained. This provides a rapid assessment of relative antiviral efficacy and so can be used to characterize dose-response relationships in real time and thereby inform therapeutic practice. Regulatory authority and treatment guideline decisions should incorporate in vivo antiviral efficacy.

**Table 1.** Summary of Patient Characteristics Included in the 2 Modified Intention-to-Treat Populations (N = 131)

Characteristic	Remdesivir	No Study Drug
No. of patients	67	69
Age, y, mean (SD)	30.1 (8.2)	30.1 (6.5)
Days since symptom onset, mean (SD)	2.4 (0.8)	2.2 (0.7)
Baseline viral load, log_10_ copies/mL, mean (range)	5.5 (2.7–7.4)	5.5 (3.0–7.9)
Vaccine doses received previously, median (range)	3 (0–4)	3 (0–4)
Antibody positive from rapid test, %^a^ (Thailand only)	90	94
Male sex, %	48	42
Sites, No.		
HTD	54	57
BP	2	2
VJ	2	3
UFMG	9	7

Abbreviations: BP, Bangplee Hospital, Thailand; HTD, Hospital for Tropical Diseases, Thailand; SD, standard deviation; UFMG, Universidade Federal de Minas Gerais, Brazil; VJ, Vajira Hospital, Thailand.

^a^Defined as immunoglobulin M or immunoglobulin G present on the rapid antibody test (BIOSYNEX COVID-19 BSS IgM/IgG, Illkirch-Graffenstaden, France) used as per manufacturer's instructions.

## Supplementary Data

Supplementary materials are available at *The Journal of Infectious Diseases* online. Consisting of data provided by the authors to benefit the reader, the posted materials are not copyedited and are the sole responsibility of the authors, so questions or comments should be addressed to the corresponding author.

## Supplementary Material

jiad275_Supplementary_Data

## Notes

***Acknowledgments.*** We thank the patients with COVID-19 who volunteered to be part of the study. We thank the DSMB (Tim Peto, Andre Siqueira, and Panisadee Avirutnan); the trial steering committee (Nathalie Strub-Wourgaft, Martin Llewelyn, Deborah Waller, and Attavit Asavisanu); Sompob Saralamba and Tanaphum Wichaita for developing the RShiny randomization app; and Mavuto Mukaka for invaluable statistical support. We also thank the staff of the Clinical Trials Unit at MORU, the staff of Thermo Fisher for their excellent support with this project, and all the hospital staff at the Hospital of Tropical Diseases, Bangplee Hospital, and Vajira Hospitals, as well as those involved in sample processing in MORU and the processing and analysis at the Faculty of Tropical Medicine molecular genetics laboratory. We thank the MORU Clinical Trials Support Group for data management and logistics, the purchasing, administration, and support staff at MORU, and those at the Brazil site who provided expert help in managing patients (Joseane Fratari, Josiane Vaz, Fátima Brant, and Lísia Esper).

***Disclaimer.*** The funders had no role in the design, conduct, analysis, or interpretation of the trial.

***Financial support.*** The PLATCOV study is supported by the Wellcome Trust (grant number 223195/Z/21/Z) through the COVID-19 Therapeutics Accelerator. The infrastructure in the Brazilian site was partially supported by funds from the National Institute of Science and Technology in dengue and host–microbial interactions (grant number MCTI/CNPQ/CAPES/FAPEMIG 465425/2014-3). Funding to pay the Open Access publication charges for this article was provided by MORU.
